# Visceral Adiposity and Its Impact on Nephrolithiasis: A Narrative Review

**DOI:** 10.3390/jcm13144065

**Published:** 2024-07-11

**Authors:** Carlo Augusto Mallio, Laura Cea, Valerio D’Andrea, Andrea Buoso, Caterina Bernetti, Bruno Beomonte Zobel, Federico Greco

**Affiliations:** 1 Fondazione Policlinico Universitario Campus Bio-Medico, Via Alvaro del Portillo, 200, 00128 Roma, Italy; c.mallio@policlinicocampus.it (C.A.M.); laura.cea@unicampus.it (L.C.); valerio.dandrea@unicampus.it (V.D.); andrea.buoso@unicampus.it (A.B.); c.bernetti@policlinicocampus.it (C.B.); b.zobel@policlinicocampus.it (B.B.Z.); 2 Research Unit of Radiology, Department of Medicine and Surgery, Università Campus Bio-Medico di Roma, Via Alvaro del Portillo, 21, 00128 Roma, Italy; 3 Department of Radiology, Cittadella della Salute, Azienda Sanitaria Locale di Lecce, Piazza Filippo Bottazzi, 2, 73100 Lecce, Italy

**Keywords:** adipose tissue, body mass index, kidney stones, nephrolithiasis, visceral fat, visceral adiposity index, computed tomography

## Abstract

Kidney stones represent a serious medical problem, resulting from several factors such as diet, genetics, and certain medical conditions. Visceral adipose tissue has been shown in recent research to play a significant role in kidney stone formation, making it a more precise indicator than traditional obesity indicators such as body mass index. The main aim of this review is to summarize studies on visceral obesity as a predictive marker for nephrolithiasis and to highlight new mechanistic pathways such as adipokine-mediated inflammation and its impact on kidney stone formation. This review emphasizes the importance of considering visceral fat in the prevention and management of kidney stones, suggesting that targeted strategies to reduce visceral fat could decrease the incidence of kidney stones and their management costs. Further prospective studies are needed to validate these findings and propose preventive strategies based on visceral adiposity assessments.

## 1. Introduction

Kidney stones, or nephrolithiasis, constitute crystalline aggregates within the urinary system [1]. The prevalence of urinary lithiasis in developed countries is about 5%, and its symptomatic onset (renal colic) serves as the main reason for emergency room visits due to urological issues [1,2].

There are various mechanisms leading to the formation of lithiasis, among them [3]:

- Saturation: characterized by an increase in the urine concentration of a substance, potentially leading to the formation of stones;
- Alteration in urinary pH: for instance, a high pH promotes the formation of calcium phosphate stones, while a decrease in pH favors uric acid lithiasis [4];
- Modification of crystallization inhibitors;
- An increase in lithogenic substances.

There are several types of lithiasis (Table 1), such as calcium lithiasis, which is the most common form, accounting for about 70% [5]. There are two types of calcium-based stones: calcium oxalate (more common) and calcium phosphate [6].

Uric acid stones rank second in frequency, comprising about 10–15% of cases. Their unique composition renders them radiotransparent in X-ray evaluations [7].

Less frequent types of stones include infectious and cystine lithiasis.

Infectious lithiasis, responsible for about 10% of cases, occurs due to colonization of the urinary tract by urease-positive germs (e.g., Proteus, Pseudomonas, Klebsiella, Serratia or Enterobacter) that convert urea into ammonia and carbon dioxide. Ammonia is then hydrolyzed, resulting in an increase in pH and subsequent formation of struvite stones [8]. 

Cystine stones, making up 1% of cases, are typically associated with cystinuria, an autosomal recessive disease [9].

Renal colic typically manifests as a low back pain of a colic nature, which may radiate to the genital region. It can be accompanied by additional symptoms, such as nausea, vomiting, dysuria, pollakiuria, tenesmus and hematuria [10]. The diagnosis is facilitated by abdominal ultrasound, which assesses potential dilation in the calico-pyelic cavities or the possible presence of lithiasis in the upper urinary tract or in the bladder. However, non-contrast computed tomography (CT) remains the diagnostic gold standard [11].

In non-complicated cases, treatment is generally conservative, involving hydration, analgesics (excluding spasmolytics) and non-steroidal anti-inflammatory drugs to reduce local edema. For distal lithiasis, alpha-blockers may also be considered [12,13]. 

In more complex cases, such as in cases of infection or severe hydroureteronephrosis, careful monitoring along with antibiotics may be necessary [14]. Interventions such as a double-J catheter or percutaneous nephrostomy could also be required [15,16]. 

For stones of significant size (peri-centimetric), spontaneous expulsion is unlikely; hence, extracorporeal shock wave lithotripsy (ESWL) may be necessary. Stones greater than 2 cm could even necessitate open or percutaneous surgery to reduce the lithiasic mass associated with ESWL for lithiasic remains [17,18]. 

The significant increase in renal colic cases presenting in emergency settings, combined with the high cost associated with hospitalization and treatment, underlines the importance of prevention [19]. Several risk factors contributing to kidney stone formation have been identified, including genetic predisposition, eating habits and regular intake of certain medications or supplements (such as diuretics and calcium-based antacids), but also conditions such as gout, chronic kidney disease and obesity [20,21,22].

Recent research studies have directed their focus towards visceral fat (VF) as a key factor in stone formation. VF is deemed a more accurate indicator than general obesity or body mass index (BMI) since it is more closely related to metabolic and inflammatory disorders that can alter urine composition and thus affect stone formation [23].

Moreover, VF is also associated with several metabolic disorders, including insulin resistance, diabetes mellitus type 2 and dyslipidemia [24,25].

**Figure 1:**
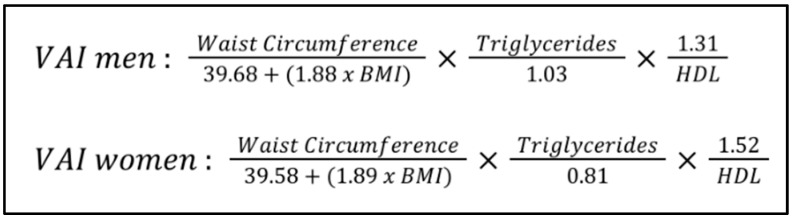
Formula used to calculate the Visceral Adiposity Index (VAI).

The Visceral adiposity Index (VAI) is a mathematical model that incorporates waist circumference, BMI, triglyceride levels and high-density lipoprotein (HDL) cholesterol levels to estimate VF distribution and its associated metabolic risk [26]. It is derived from a combination of anthropometric and metabolic parameters, including waist-to-hip ratio, BMI, plasma triglycerides and HDL cholesterol level [27] (Figure 1).

The measurement units are the following:

- Waist circumference is measured in centimeters (cm);
- BMI is a unitless measurement, as it is calculated as weight in kilograms (kg) divided by the square of height in meters (m^2^);
- Triglycerides are measured in millimoles per liter (mmol/L);
- HDL (is also measured in millimoles per liter (mmol/L).

This formula has been developed to better reflect the metabolic risk posed by VF accumulation, overcoming the limitations of individual indicators and also accounting for gender-specific variations. 

To determine which factor affects the VAI value the most, sensitivity analysis must be performed. This involves changing each factor individually and observing the effect on the VAI value. In general terms:

Waist circumference and BMI: since both are components related to adiposity, changes in these values can significantly impact the VAI, particularly waist circumference as it is directly related to VF.

Triglycerides and HDL: these are lipid parameters. High levels of triglycerides or low levels of HDL cholesterol can increase the VAI.

Given the formula, it is likely that waist circumference might have the most direct and significant impact on the VAI due to its direct relationship with VF. However, the exact factor with the most impact can vary depending on individual variations and initial values of the factors.

This review aims to gather and analyze the available scientific literature to consolidate the evidence on the association between visceral obesity and nephrolithiasis, highlighting new biomarkers and mechanistic findings.

## 2. Methods

The literature research was performed on April 2024 using MEDLINE PubMed Central and Google Scholar, taking into account only articles written in English and without limits of time span. Keywords used for the search included “kidney stones”, “nephrolithiasis”, “visceral fat”, “visceral adiposity index” and “computed tomography”. Relevant articles related to VF and kidney stones were selected and included.

## 3. Discussion

Seven studies have been selected for this review (Table 2). All are retrospective studies aimed at evaluating a correlation between VF and the development of kidney stones. 

The VAI has been identified as an indicator with higher sensitivity and specificity compared to commonly used metrics for assessing metabolic disorders, such as waist circumference, BMI and blood lipids [28,29] (Table 3).

**Table 2:** Summary of the main studies on the link between VF and kidney stones, detailing authors, year, study aims, patient numbers and key findings. Abbreviations: CT (Computed Tomography), VAI (Visceral Adiposity Index), VF (Visceral Fat), VSR (Visceral Fat-to-Subcutaneous Fat Ratio).

Authors Year	Aim/Rationale	Patients (n°)	Conclusions
Hou et al., 2022 [30]	To explore the potential of the VAI as an indicator of the risk of kidney stones.	59,842	The VAI was positively correlated with the prevalence of kidney stones, suggesting that a higher VAI is associated with a higher risk of developing kidney stones.
Sönmez et al., 2022 [31]	To assess the relationship between the VAI and nephrolithiasis in patients who underwent surgery for kidney stones.	148	A significant relationship was detected between nephrolithiasis and the VAI, indicating its importance as a new gender-specific metabolic index.
Wang et al., 2022 [32]	To investigate the association between the VAI and kidney stone incidence in an American adult population.	13,871	The VAI was positively correlated with kidney stones, and the association was significant, suggesting a higher VAI increases the risk of nephrolithiasis.
Bartani et al., 2017 [33]	To investigate the relationship between nephrolithiasis and body fat, particularly the VSR, in obese individuals.	110	VF and subcutaneous fat, as well as the VSR, were identified as important risk factors for kidney stone formation.
Akarken et al., 2015 [34]	To examine the relationship between stone disease and the amount of VF, as measured with unenhanced CT.	288	The ratio of VF to subcutaneous fat, along with obesity, hyperlipidemia and hypertension, was identified as a new factor in the formation of kidney stones.
Liang et al., 2023 [35]	To evaluate the association between the VAI and the risk of kidney stone formation and recurrence.	9886	The VAI showed a positive association with the risk of kidney stone recurrence, particularly in participants with diabetes, suggesting the influence of metabolic factors on kidney stone pathology.
Zhou et al., 2013 [36]	To explore the effects of VF area and other metabolic parameters on kidney stone composition in patients who underwent percutaneous nephrolithotomy.	269	Visceral adiposity, as measured by the VF area on CT scans, is associated with uric acid nephrolithiasis, highlighting the need for considering VF in the management and prevention of kidney stones.

Although obesity remains an important factor involved in the high prevalence of urolithiasis [37], existing obesity indicators lack the precision needed to accurately predict the risk of kidney stone development within the population. 

A high VAI significantly correlates with an increased risk of kidney stones, outperforming other risk factors statistically.

VF, being metabolically active, secretes various substances, including proinflammatory cytokines, adipokines and hormones [38,39]. These substances influence urinary metabolism and pH, potentially altering urine composition, by increasing the concentration of calcium, oxalate and uric acid, which are the main constituents of kidney stones [40,41]. Furthermore, VF is linked with insulin resistance, which may enhance renal calcium reabsorption and reduce urinary citrate levels, a crucial inhibitor of stone formation [42].

**Figure 2:**
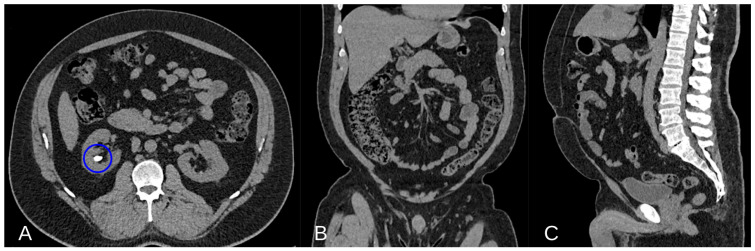
Non-contrast Axial CT scan image demonstrating a calcium lithiasis in a caliceal group of the right kidney (blue circle in (**A**)). Non-contrast Coronal and Sagittal CT scan showing a good representation of VF ((**B**,**C**) respectively).

Epidemiological studies support the higher risk of kidney stone development in individuals with elevated VF, highlighting the need to consider VF not only as a risk factor for metabolic and cardiovascular diseases but also for nephrolithiasis [43] (Figure 2).

Limited major studies exist on this subject; among them, three utilized the National Health and Nutrition Examination Survey (NHANES) datasets, an American research initiative aiming to compile health and nutrition data of the U.S. population through interviews and physical examinations.

For instance, Wang et al. [32] conducted a retrospective analysis using cross-sectional data collected from 2007 to 2018, encompassing a total of 13,871 American adults over 20 years old with nephrolithiasis. The study divided patients into four groups according to VAI quartiles: Q1 (11.96–42.89), Q2 (42.90–74.45), Q3 (74.45–131.43) and Q4 (131.45–611.34). The mean standard deviations of the VAI in the four groups are Q1 (29.07 ± 8.22), Q2 (57.53 ± 8.81), Q3 (99.52 ± 16.25) and Q4 (225.92 ± 95.83). The association between the VAI and nephrolithiasis was evaluated using logistic regression models, both unadjusted and adjusted for various potential confounders. Model 1 was unadjusted. Model 2 was adjusted for age, gender and race. Model 3 included adjustments for gender, age, race, education, marital status, poverty–income ratio, smoking, alcohol consumption, high blood pressure, diabetes, congestive hearth failure, cancer, HEI2015 total score, energy intake, vigorous activity and moderate activity.

The multivariate regression analyses, adjusted for different confounders, demonstrated a positive correlation between the VAI and kidney stones. In model 1, the odds ratio (OR) was 1.002 (95% CI: 1.001–1.003); in model 2, it was 1.002 (95% CI: 1.001–1.002); and in model 3, it was 1.001 (95% CI: 1.000–1.001). Additionally, participants in the highest quartile (Q4: 131.45–611.34) had a significantly increased risk of developing kidney stones compared to those in the lowest quartile (Q1). The ORs were 1.879 (95% CI: 1.589–2.222) in model 1, 1.644 (95% CI: 1.384–1.953) in model 2 and 1.329 (95% CI: 1.104–1.600) in model 3. The *p*-value for the trend across all three models was less than 0.05. After adjusting for other variables, no significant interactions were observed. Moreover, the association between the VAI and kidney stones was examined using generalized additive models, smooth curve fitting, and piecewise linear regression. The results of the fully adjusted model revealed a curved relationship between the VAI and kidney stone incidence. As the VAI increased, the risk of developing nephrolithiasis rose parabolically and then gradually leveled off after the VAI reached a certain value. The piecewise linear regression used to identify the inflection point showed that when the VAI was below 75.130, each unit increase in the VAI raised the risk of developing kidney stones [OR: 1.005 (95% CI: 1.001–1.009)], and when the VAI was above 75.130, the risk remained steady [OR: 1.000 (95% CI: 1.000–1.001)]. The *p*-value for the likelihood ratio test was less than 0.05, indicating a nonlinear association between the VAI and kidney stones. According to the piecewise linear regression analysis, when the VAI was <75.130, there was a positive and approximately linear correlation between the VAI and the risk of kidney stones (*p* = 0.0084). The results reveal a clear relationship between the VAI and the prevalence of kidney stones, theoretically supporting the use of the VAI to assess the potential risk of developing kidney stones [32].

The study with the largest cohort was conducted by Hou et al. [30], which gathered a total of 29,384 participants over 20 years old from 2007 to 2018, using NHANES data and using the same covariants of a previous study. The results reported 2781 participants with kidney stones, with logistic regression analysis demonstrating a higher average VAI among patients with a history of kidney stones compared to the control group. In particular, the VAI was 0.74 (0.70, 0.78) in the kidney stones group and 0.55 (0.52, 0.57) in the control group. After adjusting for confounders, the prevalence of kidney stones increased by 13% for each unit increase in the VAI (OR = 1.13, 95% CI: 1.08, 1.19). Additionally, a linear relationship was observed between the VAI and kidney stone prevalence. Subgroup analysis revealed a positive correlation between the VAI and kidney stone risk in both males (OR = 1.14, 95% CI: 1.07, 1.22) and females (OR = 1.14, 95% CI: 1.05, 1.24), whites (OR = 1.20, 95% CI: 1.11, 1.28) and other races, all age subgroups and non-hypertensive (OR = 1.06, 95% CI: 1.08, 1.25), and non-diabetic subgroups (OR = 1.14, 95% CI: 1.07, 1.21). An incremental increase in the VAI was associated with a 13% rise in kidney stone prevalence. This positive correlation was consistent across several subgroups, suggesting an increased risk of kidney stones with higher VAI levels [30].

The most recent study utilizing NHANES data was performed by Liang et al. [35], spanning from 2007 to 2014, and included 9886 participants who suffered at least once in their lives from nephrolithiasis. 

Continuous variables were reported as means with standard errors, while categorical variables were reported as proportions. Differences between subjects grouped by VAI quartiles were assessed using a weighted Student’s *t*-test for continuous variables and a weighted Chi-Square test for categorical variables. To explore the association between the VAI and the risk of kidney stones and their recurrence, three models of multivariable logistic regression were used. Model 1 had no covariate adjustments. Model 2 adjusted for gender, age, race, educational level and BMI (as a continuous variable). Model 3 further adjusted for gender, age, race, educational level, BMI (as a continuous variable), physical activity, diabetes and hypertension. Subgroup analysis was performed to examine the correlation between the VAI and the risk of kidney stones and their recurrence across different stratification factors.

The average VAI index was 2.09 ± 0.04. The overall incidence of kidney stones was 9.24%, increasing with higher VAI quartiles (Quartile 1: 6.08%, Quartile 2: 8.57%, Quartile 3: 9.95%, Quartile 4: 12.40%). The recurrence rate of kidney stones among all subjects was 2.97%, with higher VAI quartiles associated with a greater risk of recurrence (Quartile 1: 1.78%, Quartile 2: 1.86%, Quartile 3: 3.78%, Quartile 4: 4.51%). Statistically significant differences were observed among the four VAI quartiles in terms of age, total cholesterol, HDL cholesterol, triglycerides, waist circumference, race, poverty–income ratio, education, physical activity, BMI, alcohol use, smoking status, hypertension and diabetes mellitus (*p* < 0.05). Compared to the lowest VAI quartile, individuals in higher VAI quartiles had higher levels of total cholesterol, triglycerides, waist circumference, BMI, lower levels of HDL cholesterol, and a higher likelihood of developing hypertension and diabetes. Additionally, participants in higher VAI quartiles were more often Mexican-American and Non-Hispanic white, and were less physically active.

Once again, statistical analysis underscored a significant correlation between an increased VAI and the incidence of kidney stones and recurrences in the American population, irrespective of personal factors such as gender, age or health status [35].

The three studies mentioned above used the NHANES datasets and the specific value of the VAI to catalog and compare their patients. 

Other studies have employed different methodologies to quantify peri-visceral fat, such as CT scans. For instance, Zhou et al. [36] found that individuals with uric acid stones had higher rates of hypertension and greater VF area.

This study included 269 patients undergoing percutaneous nephrolithotomy. VF area was measured from a single 5 mm axial slice at the umbilical level using the Aquarius iNtuition computerized fat analysis tool, version 4.4.6.100.2862. VF was identified using a fixed attenuation range of −190 to −30 HUs, consistent with previous studies [44,45]. Out of the 269 patients included in the final analysis, 220 (81.8%) had non-uremic acid (UA) stones and 49 (18.2%) had UA stones as part or all of the stone composition. In univariate analysis, patients with UA stones exhibited significantly higher rates of hypertension (67.4% vs. 47.3%, *p* = 0.011) and coronary artery disease (14.3% vs. 4.6%, *p* = 0.011) compared to those with non-UA stones. Moreover, a significantly higher mean VF area was seen in patients with UA stones (209.3 vs. 161.9 cm^2^, *p* = 0.0013). A significantly greater percentage of patients with UA stones had a high VF area compared to non-UA stone patients (59.2% vs. 34.1%, *p* = 0.003). Logistic regression analysis was conducted to examine the association between UA stones and the three VF area categories (low, medium and high), as well as hypertension and coronary artery disease, which were individually associated with UA stones in univariate analysis. In this model, only a high VF area was significantly associated with UA stones (OR 3.12, 95% CI 1.25–7.76, *p* = 0.015), while medium VF area, hypertension and coronary artery disease were not.

In the logistic regression analysis, including patient characteristics (age, gender and BMI) to determine potential confounding by these variables, the VF area was divided into tertiles: low (less than 124 cm^2^), medium (124 cm^2^ to 186.9 cm^2^) and high (187 cm^2^ or greater). Compared to the low VF area group, hypertension (OR 2.16, 95% CI 1.05–4.45, *p* = 0.04) and high VF area (OR 3.64, 95% CI 1.22–10.85, *p* = 0.02) emerged as independent predictors of UA nephrolithiasis, while medium VF area and coronary artery disease were not significant predictors.

When multivariate analysis was repeated in the subset of patients with available serum UA, hypertension and high VF area were no longer significant. 

Patients with UA stones exhibited a significantly larger mean VF area, with a high VF area (greater than 187 cm^2^) as an independent risk factor for UA nephrolithiasis [36].

Akarken et al. [34] involved 149 kidney stone patients identified through CT scans from August 2012 to April 2013, while the control group comprised 139 individuals who reported flank pain but had no CT evidence of nephrolithiasis. Patients were categorized by age, sex, BMI, VF and subcutaneous fat areas and serum level of low-density lipoproteins and triglycerides. VF area and subcutaneous fat area were measured on a single CT axial image located at the level of the umbilicus, by determining the values between −190 and −30 HUs as fixed attenuation range, using the Aquarius iNtuition computerized fat analysis tool, version 4.4.6.100.2862 [44,46].

The respective measurements for the stone and control groups were as follows: BMI (29.1 vs. 27.6 kg/m^2^), VF area (186.0 vs. 120.2 cm^2^) and subcutaneous fat area (275.9 vs. 261.9 cm^2^), with *p*-values of 0.01, 0.01 and 0.36, respectively. Multivariate analysis identified hyperlipidemia (*p* = 0.003), hypertension (*p* = 0.001) and the VF area % (*p* = 0.01) as factors increasing the risk of kidney stone formation [34].

Sönmez et al. [31] investigated the variation in VAI between individuals with nephrolithiasis (103 patients) and a control group (45 patients), aiming to also assess the association between the VAI, stone characteristics and surgical outcomes. 

Their prospective study encompassed patients undergoing percutaneous nephrolithotomy (PCNL) (45.6%) and intrarenal retrograde surgery (RIRS) (54.4%) for kidney stones in the same urology department between January 2017 and December 2019. 

The control group consisted of healthy individuals without documented lithiasis on a CT scan, residing in the same geographical area with similar nutritional habits as the patient group. Exclusion criteria were individuals under 18 years old, smokers, cancer patients or those with urinary tract diseases other than stones (including kidney failure).

Various data were taken into consideration, such as demographic and metabolic information, stone type and location, as well as surgical details and post-operative outcomes. 

VAI, waist circumference and BMI values showed significantly higher values in the nephrolithiasis group compared to the control group (*p* = 0.02, *p* = 0.04, *p* < 0.001, respectively).

In the multivariate analysis, among the various metabolic parameters, only the VAI was statistically significant {1.52 OR [(95% CI 1.21–1.9)], *p* < 0.001}; however, no significant relationship was found between the VAI and surgical parameters [31].

Bartani et al. [33], in 2017, broadened their research to include not only VAT but also SAT, proposing that the VAT/SAT ratio might influence kidney stone development. Their study included 103 obese individuals, equally divided into a group with a history of kidney stones and a control group without such history or other pathologies. Using bioimpedance analysis to assess body composition, researchers discovered that the VF/subcutaneous fat ratio was significantly higher in the kidney stone group (*p* = 0.012) [33]. 

The studies reviewed provide several advantages in understanding the correlation between VF and nephrolithiasis. They collectively demonstrate that the VAI is a more sensitive and specific indicator of metabolic disorders compared to traditional metrics like BMI and waist circumference. Retrospective analyses, particularly those using large datasets like NHANES, offer robust evidence supporting the positive correlation between the VAI and kidney stones, highlighting a nonlinear relationship where risk increases significantly up to a certain VAI threshold. 

Visceral adiposity contributes to nephrolithiasis through multiple mechanisms. Adipokines secreted by VF induce systemic inflammation, which in turn affects renal function and promotes stone formation. Furthermore, insulin resistance, commonly associated with VF, leads to increased renal calcium reabsorption and decreased urinary citrate excretion, both of which are critical factors in stone pathogenesis.

Studies employing various methodologies, such as CT scans and bioimpedance analysis, further validate the association by demonstrating higher VF in patients with kidney stones compared to controls. These comprehensive analyses underscore the importance of considering VF in the risk assessment and management of nephrolithiasis, offering a potential for improved predictive accuracy over traditional obesity metrics.

## 4. Conclusions

The assessment of VF emerges as a potent risk indicator, poised to transform preventive strategies. This proactive approach facilitates early detection, enabling more effective management. Identifying individuals at high risk before symptom onset can substantially reduce the incidence of kidney colic and the subsequent need for emergency room interventions. Moreover, prioritizing early intervention could significantly decrease the necessity for surgical procedures and the associated risk of complications. This comprehensive approach underscores the importance of integrating perivisceral fat evaluation into health assessments, aiming to improve the prevention and management of kidney stones, ultimately enhancing patient outcomes and reducing healthcare costs tied to advanced urolithiasis treatments.

Undoubtedly, more prospective studies are essential to validate these findings.

## Figures and Tables

**Table 1:** Summary of the main categories of urinary calculi divided according to their frequency, X-ray imaging characteristics, urine pH and treatments.

Types of Lithiasis	Frequency (%)	X-ray Opacity	pH	Treatment
Calcium	70	Radiopaque	Alkaline	Necessary to act on the cause: Primary hypercalciuria: thiazides; Hypocitraturia: citrate; Primary hyperoxaluria: pyridoxine.
Infectious (struveite)	5–10	Radiopaque	Alkaline	Antibiotics and urease inhibitors (such as propionic acid and acetoidrossamic)
Uric acid	10–15	Radiotransparent	Acid	Allopurinol (alkalized urine)
Cystine	1	Partially radiopaque	Acid	D-penicillamine (alkalized urine)

**Table 3:** Main indicators for body fat assessment with their characteristics. Abbreviations: A (advantages), BIA (bioelectrical impedance analysis), BMI (Body Mass Index), CT (Computed Tomography), D (disadvantages), MRI (Magnetic Resonance Imaging), VAI (Visceral Adiposity Index), WHR (Waist-to-Hip Ratio).

Indicators	Characteristic	Advantages (A) and Disadvantages (D)
BMI	Weight (kg) divided by the square of height (m)	A: Simplicity, speed and widespread use D: Does not distinguish between fat and lean mass; does not account for individual factors, which can influence body fat distribution
Waist Circumference	Circumference around the abdomen	A: Simplicity and speed D: Accuracy can vary depending on who performs the measurement and the technique used; does not assess body composition
Skinfold Thickness	Thickness of skinfolds measured with a caliper	A: Cost-effective and accessible D: Limited to subcutaneous fat; technique-sensitive
WHR	Ratio of waist circumference to hip circumference	A: Simple and quick; predictive of health risks D: Provides information about fat distribution rather than total body fat percentage; influenced by other factors (body shape and posture)
BIA	Body resistance to electrical current	A: Provides body composition analysis D: Results can vary depending on hydration status or metabolic conditions
MRI	Qualitative evaluation of the fat tissue	A: Highly detailed images; non-invasive and safe D: Availability; costly and time-consuming
CT	Qualitative evaluation of the body and fat tissue	A: Accurate measurement of fat; quicker than MRI D: Radiation exposure
VAI	Use a formula (Figure 1)	A: Assesses a comprehensive metabolic risk; non-invasive D: Indirect measurement
